# Maintenance pemetrexed after platinum pemetrexed chemotherapy and survival outcomes in malignant pleural mesothelioma in a multicenter real world study

**DOI:** 10.1038/s41598-026-61625-0

**Published:** 2026-07-15

**Authors:** Okan Aydin, Zehra Sucuoglu Isleyen, Mert Erciyestepe, Ahmet Emin Ozturk, Sermin Dinc Sonusen, Tugay Atasever, Simay Cokgezer, Ebru Cicek, Ayse Gul Satman Durmaz, Lamia Seker Can, Gozde Balkaya Aykut, Tuba Baydas, Miray Aydogan, Seval Ay Ersoy, Muhammed Mustafa Atci, Kayhan Erturk

**Affiliations:** 1 Department of Medical Oncology, University of Health Sciences, Prof. Dr. Cemil Tascıoglu City Hospital, Istanbul, Turkey; 2 Department of Medical Oncology, Institute of Oncology, Istanbul University, Istanbul, Turkey; 3 Department of Internal Medicine, Division of Medical Oncology, Cerrahpasa Faculty of Medicine, Istanbul University-Cerrahpasa, Istanbul, Turkey; 4 Department of Medical Oncology, University of Health Science, Bakirkoy Dr. Sadi Konuk Training and Research Hospital, Istanbul, Turkey; 5 Department of Medical Oncology, Bezmialem Vakif University Hospital, Istanbul, Turkey; 6 Department of Medical Oncology, University of Health Science, Umraniye Training and Research Hospital, Istanbul, Turkey; 7 Department of Internal Medicine, Division of Medical Oncology, Faculty of Medicine, Istanbul Medeniyet University, Istanbul, Turkey; 8 Department of Medical Oncology, University of Health Science, Kartal Dr. Lütfi Kirdar City Hospital, Istanbul, Turkey; 9 Department of Medical Oncology, Istinye University Faculty of Medicine, Liv Hospital, Istanbul, Turkey

**Keywords:** Malignant pleural mesothelioma, Maintenance therapy, Pemetrexed, Platinum-based chemotherapy, Real-world study, Cancer, Oncology

## Abstract

Malignant pleural mesothelioma (MPM) is an aggressive malignancy with poor prognosis, and the role of maintenance therapy after first-line platinum–pemetrexed chemotherapy remains unclear. We conducted a multicenter retrospective study including 96 patients with metastatic or unresectable MPM treated across eight tertiary oncology centers. Eligible patients had received at least four cycles of first-line platinum–pemetrexed and achieved disease control, after which they were either administered maintenance pemetrexed (*n* = 54) or observed without further treatment (*n* = 42). Maintenance pemetrexed was associated with significantly prolonged median progression-free survival (15.5 vs. 7.9 months; *p* < 0.001) and overall survival (22.3 vs. 16.4 months; *p* = 0.002) compared with observation. Multivariate analyses confirmed maintenance pemetrexed as an independent predictor of improved progression-free survival (HR 2.92; 95% CI 1.81–4.72; *p* < 0.001) and overall survival (HR 2.10; 95% CI 1.24–3.55; *p* = 0.005). Non-epithelioid histology was independently associated with worse outcomes. Subgroup analyses demonstrated that the survival benefit of maintenance pemetrexed persisted in patients achieving both partial response and stable disease after first-line chemotherapy. Importantly, maintenance therapy was well tolerated, with no discontinuations due to toxicity, highlighting its feasibility in routine practice. These real-world findings suggest that maintenance pemetrexed may be associated with improved survival outcomes in patients with MPM who achieve disease control after first-line therapy.

**Supplementary Information:**

The online version contains supplementary material available at 10.1038/s41598-026-61625-0.

## Introduction

Malignant pleural mesothelioma (MPM) is a rare but aggressive malignancy arising from the mesothelial cells of the pleura and accounts for approximately 0.3% of all malignant tumors^1^. The epidemiology and pathogenesis of MPM are strongly linked to asbestos exposure, which drives chronic inflammation, genetic alterations, and malignant transformation of mesothelial cells^2^. The majority of patients are diagnosed at an advanced stage of disease, at which point the prognosis is particularly poor^3^. Despite advances in diagnostic techniques and systemic therapies, patients with advanced-stage MPM have a dismal clinical outcome, with a median overall survival not exceeding 18 months^4^.

Malignant pleural mesothelioma is histologically classified into three main subtypes: epithelioid, sarcomatoid, and biphasic (mixed). The epithelioid subtype is the most frequent, representing approximately 60% of cases, while sarcomatoid and biphasic subtypes each account for about 20% of diagnoses. These histological subtypes are clinically meaningful, as they are associated with distinct tumor biology and prognostic outcomes^5^.

In patients with unresectable malignant pleural mesothelioma, the combination of pemetrexed and cisplatin is an established first-line systemic treatment. In a pivotal randomized phase III trial, pemetrexed plus cisplatin demonstrated significantly superior outcomes compared with cisplatin alone, and based on these findings, platinum–pemetrexed chemotherapy became the cornerstone of first-line treatment^6^. Carboplatin may be used as an alternative to cisplatin in combination with pemetrexed for patients who are unable to tolerate cisplatin^7^.

Although platinum–pemetrexed chemotherapy remains a central component of the initial treatment of malignant pleural mesothelioma, recent advances have expanded the range of therapeutic options to include immunotherapy-based approaches. The randomised phase III CheckMate 743 trial found that first-line nivolumab plus ipilimumab significantly improved overall survival compared with platinum-based chemotherapy in patients with unresectable disease^8^. Nevertheless, platinum–pemetrexed–based regimens remain widely used in routine clinical practice, particularly in patients with the epithelioid histological subtype. In this subgroup, chemotherapy remains a guideline-recommended first-line option and is also commonly used in patients who are not suitable candidates for immunotherapy^4,9^.

Despite the ongoing use of chemotherapy as a primary treatment, the role of maintenance therapy for patients who achieve disease control remains unclear. Evidence regarding the efficacy of maintenance strategies in malignant pleural mesothelioma is limited and inconsistent, and no standard approach has been established. Consequently, decisions regarding post–first-line treatment continue to vary in routine clinical practice.

In this context, this study aimed to evaluate the clinical outcomes of malignant pleural mesothelioma patients who received pemetrexed maintenance therapy following first-line platinum–pemetrexed chemotherapy. Using real-world data, we sought to assess the effectiveness and safety of maintenance pemetrexed in patients who achieved disease control after initial systemic treatment.

## Materials and methods

This multicenter, retrospective study included data collected from eight high-volume tertiary referral centers in Istanbul. Patients diagnosed with malignant pleural mesothelioma and presenting with metastatic or unresectable disease were screened for eligibility.

Eligible patients had received at least four cycles of first-line platinum-pemetrexed chemotherapy and achieved disease control, as defined by complete response, partial response or stable disease on radiological evaluation. Tumor response was assessed based on the revised modified Response Evaluation Criteria in Solid Tumors (modified RECIST) version 1.1, developed specifically for malignant pleural mesothelioma^10^. They were then classified according to whether they received maintenance therapy with pemetrexed or were observed without treatment.

Patients in the maintenance group were required to have received at least four cycles of pemetrexed during the maintenance phase, as well as undergoing at least one radiological response assessment. Similarly, patients in the observation group were required to undergo at least one radiological evaluation during follow-up, performed approximately three months after completing first-line chemotherapy. Patients who died before the initial post-treatment imaging assessment were excluded from the analysis.

Approximately 390 patients with malignant pleural mesothelioma were screened across the participating centers during the study period. A total of 96 patients met the predefined eligibility criteria and were included in the final analysis. Of these patients, 54 received maintenance therapy with pemetrexed, while the remaining 42 were managed with observation alone following first-line chemotherapy.

Progression-free survival (PFS) was defined as the time from the initiation of first-line platinum–pemetrexed chemotherapy to the occurrence of documented disease progression or death from any cause. Overall survival (OS) was defined as the time from the initiation of first-line platinum–pemetrexed chemotherapy to death from any cause or the last follow-up.

Statistical analyses were performed using the Statistical Package for the Social Sciences (SPSS) software, version 26.0 (IBM Corp., Armonk, NY, USA). Categorical variables were expressed as frequencies and percentages, while continuous variables were summarized using median values and ranges. Comparisons between categorical variables were performed using the chi-square test or Fisher’s exact test, as appropriate. Progression-free survival (PFS) and overall survival (OS) were estimated using the Kaplan–Meier method, and differences between groups were compared using the log-rank test. Associations between clinical variables and survival outcomes were evaluated using both univariate and multivariate Cox proportional hazards regression analyses, with hazard ratios (HRs) and corresponding 95% confidence intervals (CIs) reported. In multivariate Cox regression analyses for PFS and OS, age and sex were included regardless of statistical significance, together with variables demonstrating a p value < 0.20 in univariate analysis. A two-sided p value of < 0.05 was considered statistically significant.

This study was approved by the Ethics Committee of Istanbul Prof. Dr. Cemil Tascioglu City Hospital (Approval Number: 2026/55). The requirement for informed consent was waived by the Ethics Committee of Istanbul Prof. Dr. Cemil Tascioglu City Hospital due to the retrospective nature of the study. This study was conducted in accordance with the Declaration of Helsinki, and all methods were performed in accordance with the relevant guidelines and regulations.

## Results

A total of 96 patients with malignant pleural mesothelioma were included in the analysis. Demographic and baseline clinical characteristics of the patients are described in Table 1. The mean age of the study population was 60.4 ± 10.6 years, and the median follow-up duration was 42.5 months.

In terms of histological subtypes, epithelioid mesothelioma was the most common, observed in 79 patients (82.3%). This was followed by the biphasic subtype (12.5%) and the sarcomatoid subtype (5.2%).

At baseline, one-third of the patients were considered resectable. All of these patients (*n* = 32, 33.3%) underwent surgery, including extrapleural pneumonectomy (EPP) in 13 patients (13.5%) and pleurectomy/decortication (P/D) in 19 patients (19.8%). The remaining 64 patients (66.7%) were treated with first-line platinum–pemetrexed chemotherapy for metastatic or unresectable disease.

Among the 32 surgically treated patients, adjuvant chemotherapy was administered to a proportion of patients, most commonly cisplatin plus pemetrexed (68.8%), while a smaller proportion received carboplatin plus pemetrexed (6.3%) or no adjuvant chemotherapy (25.0%). During follow-up, all surgically treated patients developed disease recurrence or metastasis at least 6 months after completion of adjuvant chemotherapy and were therefore retreated with first-line platinum–pemetrexed chemotherapy, consistent with standard clinical practice, and were included in the present analysis.

In the metastatic or unresectable setting, all 96 patients received first-line platinum–pemetrexed chemotherapy, consisting of cisplatin plus pemetrexed in 54.2% and carboplatin plus pemetrexed in 45.8%.

Radiotherapy was administered to 41 patients (42.7%) during the course of the disease, while 55 patients (57.3%) did not receive radiotherapy.

With respect to best response to first-line platinum–pemetrexed chemotherapy, no complete responses were observed. Partial response was the most frequent outcome, observed in 76.0% of patients, whereas stable disease was documented in 24.0%.

The baseline demographic and clinical characteristics of patients receiving maintenance pemetrexed were generally well balanced with those of patients not receiving maintenance therapy. No statistically significant differences were observed between the two groups with respect to age, sex, ECOG (Eastern Cooperative Oncology Group) performance status, histological subtype, resectability status, surgical approach, adjuvant treatment, initial treatment for metastatic or unresectable disease, or radiotherapy status (all *p* > 0.05). However, patients who subsequently received maintenance pemetrexed achieved a significantly higher best response to first-line platinum–pemetrexed chemotherapy than those who did not receive maintenance therapy (*p* = 0.001).

**Table 1:** Baseline demographic and clinical characteristics of patients according to maintenance pemetrexed status.

		Maintenance pemetrexed, n (%)	No maintenance pemetrexed, n (%)	All patients, n (%)	p
Sex	Woman	26 (48.1)	15 (35.7)	41 (42.7)	0.222
	Man	28 (51.9)	27 (64.3)	55 (57.3)	
Age	< 65	36 (66.7)	26 (61.9)	62 (64.6)	0.628
	≥ 65	18 (33.3)	16 (38.1)	34 (35.4)	
ECOG-PS	0	23 (42.6)	17 (40.5)	40 (41.7)	0.921
	1	20 (37.0)	15 (35.7)	35 (36.5)	
	2	11 (20.4)	10 (23.8)	21 (21.9)	
Histology	Epithelioid	47 (87.0)	32 (76.2)	79 (82.3)	0.202
	Sarcomatoid	1 (1.1)	4 (9.5)	5 (5.2)	
	Biphasic	6 (11.1)	6 (14.3)	12 (12.5)	
Resectability	Resectable	22 (40.7)	10 (23.8)	32 (33.3)	0.081
	Unresectable	32 (59.3)	32 (76.2)	64 (66.7)	
Surgical status	No surgery	32 (59.3)	32 (76.2)	64 (66.7)	0.071
	EPP	11 (20.4)	2 (4.8)	13 (13.5)	
	P/D	11 (20.4)	8 (19.0)	19 (19.8)	
Adjuvant therapy (surgically treated patients), *n* = 32	No adjuvant chemotherapy	3 (13.6)	5 (50.0)	8 (25.0)	0.071
	Cisplatin + Pemetrexed	17 (77.3)	5 (50.0)	22 (68.8)	
	Carboplatin + Pemetrexed	2 (9.1)	0 (0.0)	2 (6.3)	
First-line treatment for metastatic or unresectable disease	Cisplatin + Pemetrexed	26 (48.1)	26 (61.9)	52 (54.2)	0.180
	Carboplatin + Pemetrexed	28 (51.9)	16 (38.1)	44 (45.8)	
Radiotherapy status	Radiotherapy	26 (48.1)	15 (35.7)	41 (42.7)	0.222
	No radiotherapy	28 (51.9)	27 (64.3)	55 (57.3)	
Best response to first-line platinum–pemetrexed chemotherapy	PR	48 (88.9)	25 (59.5)	73 (76.0)	0.001
	SD	6 (11.1)	17 (40.5)	23 (24.0)	

ECOG-PS: Eastern Cooperative Oncology Group performance status, EPP: extrapleural pneumonectomy, P/D: pleurectomy/decortication, PR: partial response, SD: stable disease.

Univariate and multivariate Cox regression analyses were performed to evaluate factors associated with progression-free survival (Table 2). In univariate analysis, receipt of maintenance pemetrexed was significantly associated with prolonged progression-free survival compared with no maintenance therapy. This association remained statistically significant in multivariate analysis, identifying maintenance pemetrexed as an independent factor associated with longer progression-free survival.

In addition, best response to first-line platinum–pemetrexed chemotherapy was significantly associated with progression-free survival. Patients who achieved a partial response experienced significantly longer progression-free survival than those with stable disease, and this association persisted in multivariate analysis.

Histological subtype was also significantly associated with progression-free survival, with patients harboring non-epithelioid histology demonstrating significantly shorter progression-free survival in both univariate and multivariate analyses.

ECOG performance status was independently associated with progression-free survival. Compared with patients with ECOG performance status 0, those with poorer performance status experienced a significantly higher risk of progression in both univariate and multivariate analyses.

Other variables, including age, sex, resectability status, surgical approach, first-line chemotherapy regimen, and number of chemotherapy cycles, were not significantly associated with progression-free survival in either univariate or multivariate analyses (all *p* > 0.05).

**Table 2:** Univariate and multivariate Cox regression analyses of factors associated with progression-free survival.

Variables	Univariate analysis		Multivariate analysis	
	HR (95% Cl)	*p* value	HR (95% Cl)	*p* value
Sex Women (R) Men	1.35 (0.86–2.1)	0.191	1.34 (0.84–2.12)	0.214
Age <65 (R) ≥65	1.28 (0.81–2.03)	0.289	0.68 (0.34–1.36)	0.282
ECOG-PS 0 (R) 1 2	1.24 (0.74–2.07) 2.12 (1.21–3.71)	**0.030** 0.416 **0.009**	1.81 (1.00-3.25) 3.47 (1.50–8.03)	**0.013** 0.049 **0.004**
Histology Epithelioid (R) Non-epithelioid	2.31 (1.30–4.10)	**0.004**	1.90 (1.04–3.46)	**0.036**
Resectability Resectable (R) Unresectable	2.31 (0.77–2.02)	0.360		
Surgical status No surgery (R) EPP P/D	0.68 (0.32–1.42) 0.88 (0.50–1.54)	0.566 0.304 0.651		
First-line chemotherapy regimens Cisplatin (R) Carboplatin	1.05 (0.68–1.64)	0.817		
Number of first-line chemotherapy cycles	0.96 (0.80–1.15)	0.671		
Best response to chemotherapy PR (R) SD	2.02 (1.21–3.36)	**0.007**	1.89 (1.11–3.24)	**0.020**
Maintenance pemetrexed Yes (R) No	2.81 (1.80–4.38)	**< 0.001**	2.92 (1.81–4.72)	**< 0.001**
Radiotherapy Yes (R) No	1.13 (0.72–1.77)	0.588		

ECOG-PS: Eastern Cooperative Oncology Group performance status, EPP: extrapleural pneumonectomy, P/D: pleurectomy/decortication, PR: partial response, SD: stable disease.

Significant values are bold.

Univariate and multivariate Cox regression analyses were performed to evaluate factors associated with overall survival (Table 3). Receipt of maintenance pemetrexed was significantly associated with improved overall survival in univariate analysis, and this association remained statistically significant in the multivariate model, identifying maintenance pemetrexed as an independent factor associated with longer overall survival.

Histological subtype showed a strong association with overall survival, with non-epithelioid histology being independently associated with significantly shorter overall survival in both analyses.

ECOG performance status was independently associated with overall survival. Compared with patients with ECOG performance status 0, those with poorer performance status had a higher risk of death, with the highest mortality risk observed among patients with ECOG 2.

Radiotherapy and resectability status were significantly associated with overall survival in univariate analysis; however, these associations did not persist after adjustment in the multivariate model.

Other variables, including age, sex, surgical status, first-line chemotherapy regimen, number of chemotherapy cycles, and best response to first-line platinum–pemetrexed chemotherapy, were not significantly associated with overall survival in either univariate or multivariate analyses (all *p* > 0.05).

**Table 3:** Univariate and multivariate Cox regression analyses of factors associated with overall survival.

Variables	Univariate analysis		Multivariate analysis	
	HR (95% Cl)	*p* value	HR (95% Cl)	*p* value
Sex Women (R) Men	1.50 (0.91–2.50)	0.114	1.34 (0.78–2.28)	0.288
Age <65 (R) ≥65	1.13 (0.66–1.92)	0.660	0.93 (0.61–1.80)	0.360
ECOG-PS 0 (R) 1 2	1.22 (0.69–2.16) 2.44 (1.30–4.59)	**0.018** 0.502 **0.006**	1.61 (0.88–2.56) 6.33 (2.49–16.03)	**< 0.001** 0.125 **< 0.001**
Histology Epithelioid (R) Non-epithelioid	3.02 (1.56–5.84)	**0.001**	3.12 (1.59–6.13)	**< 0.001**
Resectability Resectable (R) Unresectable	1.76 (1.01–3.08)	**0.048**	1.67 (0.95–3.03)	0.075
Surgical status No surgery (R) EPP P/D	0.58 (0.25–1.37) 0.56 (0.29–1.09)	0.140 0.214 0.087	1.40 (0.51–3.91) 0.68 (0.29–1.18)	0.351 0.406 0.358
First-line chemotherapy regimens Cisplatin (R) Carboplatin	0.96 (0.58–1.58)	0.870		
Number of first-line chemotherapy cycles	1.01 (0.83–1.23)	0.900		
Best response to chemotherapy PR (R) SD	1.68 (0.95–2.97)	0.077	1.46 (0.79–2.70)	0.222
Maintenance pemetrexed Yes (R) No	2.20 (1.33–3.65)	**0.002**	2.10 (1.24–3.55)	**0.005**
Radiotherapy Yes (R) No	1.70 (1.01–2.88)	**0.048**	1.52 (0.75–3.06)	0.241

ECOG-PS: Eastern Cooperative Oncology Group performance status, EPP: extrapleural pneumonectomy, P/D: pleurectomy/decortication, PR: partial response, SD: stable disease.

Significant values are bold.

**Fig. 1:**
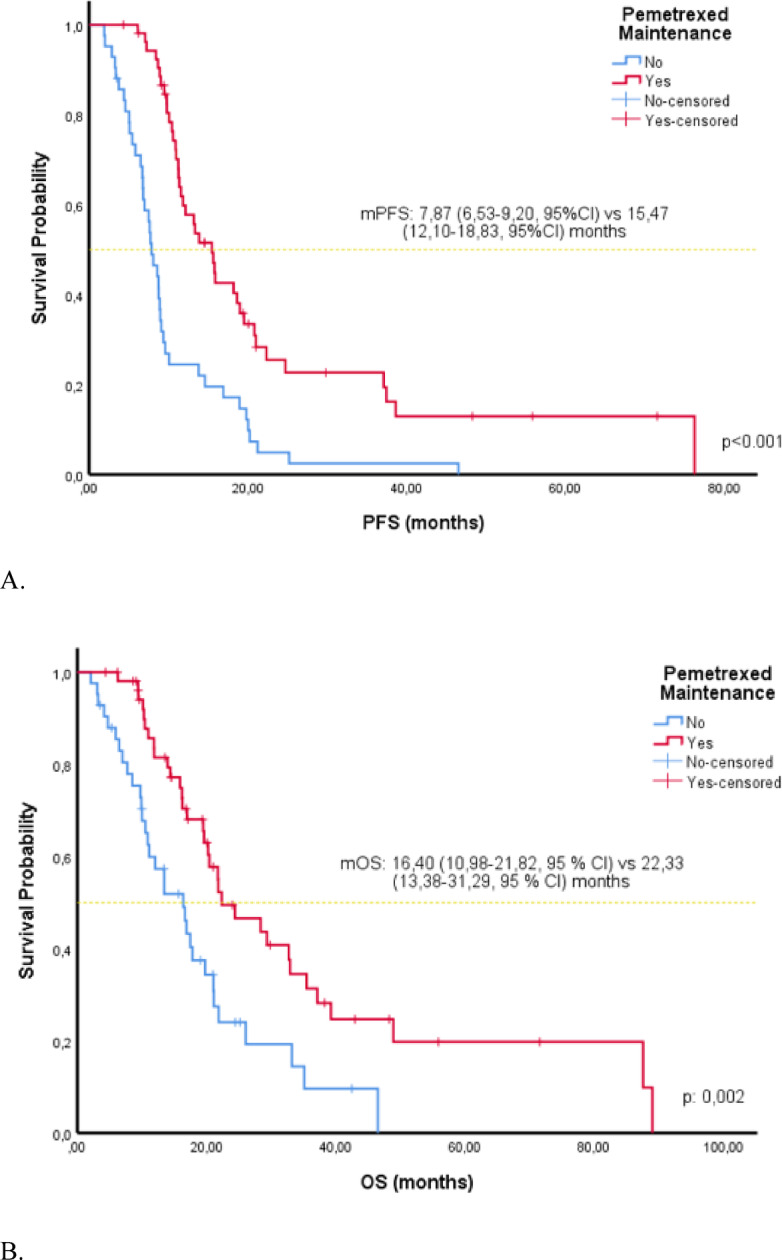
Kaplan–Meier curves for progression-free survival (**A**) and overall survival (**B**) according to maintenance pemetrexed therapy in the overall study population. mOS: median overall survival, mPFS: median progression-free survival.

Kaplan–Meier analysis showed that maintenance pemetrexed was associated with a significantly longer progression-free survival compared with no maintenance therapy, with a median PFS of 15.5 months versus 7.9 months (*p* < 0.001). Overall survival was also significantly prolonged in the maintenance pemetrexed group, with a median OS of 22.3 months compared with 16.4 months in patients who did not receive maintenance treatment (*p* = 0.002) (Fig. 1).

**Fig. 2:**
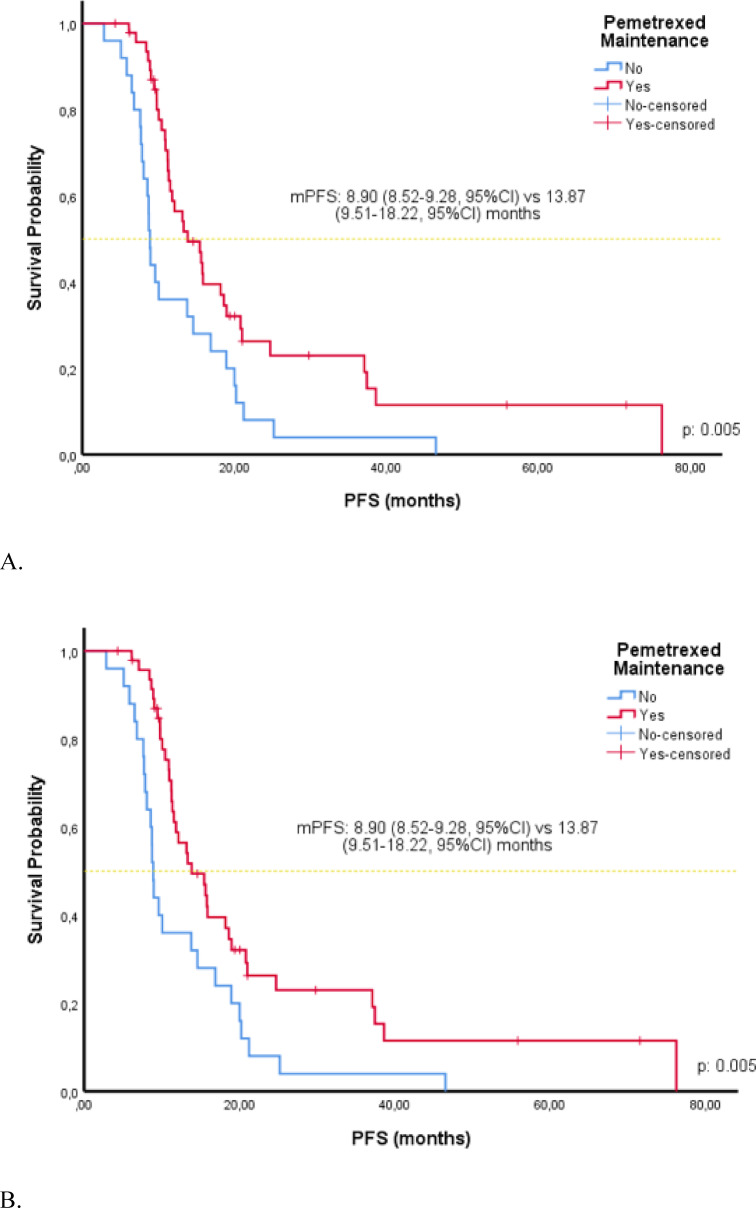
Kaplan–Meier curves for progression-free survival according to maintenance pemetrexed therapy in patients with partial response (**A**) and stable disease (**B**) after first-line platinum–pemetrexed chemotherapy. mOS: median overall survival, mPFS: median progression-free survival.

Subgroup analyses according to best response to first-line platinum–pemetrexed chemotherapy demonstrated that maintenance pemetrexed was associated with significantly prolonged progression-free survival in both response subgroups (Fig. 2). Among patients who achieved a partial response, median progression-free survival was longer in those receiving maintenance pemetrexed compared with no maintenance therapy (13.9 vs. 8.9 months; *p* = 0.005). Similarly, in patients with stable disease after first-line chemotherapy, maintenance pemetrexed was associated with a longer median progression-free survival compared with no maintenance therapy (19.5 vs. 5.4 months; *p* = 0.002).

At the time of last follow-up, 31 (32.3% of all patients) patients were alive. Among these, 16 patients had experienced disease progression, while 15 remained progression-free. Of the 15 progression-free patients, 14 were receiving maintenance pemetrexed, whereas 1 patient was under observation without maintenance therapy.

Following disease progression, post-progression treatment patterns are summarized in Supplementary Table 1. In the maintenance pemetrexed group, 11 patients (27.5%) received immunotherapy, 11 (27.5%) received chemotherapy, and 6 (15.0%) underwent platinum–pemetrexed rechallenge. In the no-maintenance group, 7 patients (17.1%) received immunotherapy, 14 (34.1%) received chemotherapy, and 3 (7.3%) underwent platinum–pemetrexed rechallenge. In addition, third-line treatment was administered to five patients in the maintenance pemetrexed group and four patients in the no-maintenance group.

During maintenance pemetrexed therapy, no patients required permanent treatment discontinuation or dose interruption due to toxicity. No clinically significant severe toxicities leading to treatment modification were observed. However, detailed toxicity grading was not systematically recorded, limiting a comprehensive assessment of lower-grade adverse events. Overall, maintenance treatment appeared to be well tolerated in routine clinical practice.

## Discussion

This multicenter real-world study included 96 patients with malignant pleural mesothelioma treated across eight high-volume tertiary oncology centers in Istanbul. In this cohort, maintenance pemetrexed therapy following first-line platinum–pemetrexed chemotherapy was associated with significantly improved progression-free survival and overall survival in patients who achieved disease control. Moreover, maintenance pemetrexed remained an independent predictor of both prolonged progression-free survival and overall survival in multivariate analyses, underscoring its potential clinical benefit beyond established patient- and disease-related prognostic factors.

Previous studies evaluating maintenance pemetrexed therapy in malignant pleural mesothelioma patients who achieved disease control after first-line chemotherapy have reported inconsistent outcomes, limiting its role in standard treatment strategies^11–14^. These discrepancies are likely related to the rarity of the disease, resulting in small sample sizes, limited statistical power, and challenges in patient accrual. In addition, differences in study design and patient selection may have further contributed to the heterogeneous results reported across studies. In this context, the phase II NVALT19 trial investigated switch-maintenance with gemcitabine after first-line platinum-based chemotherapy in patients with MPM. Although a different chemotherapeutic agent was used, the study highlighted the feasibility and potential benefit of maintenance strategies, thereby supporting the rationale for exploring pemetrexed maintenance in routine clinical practice^15^.

One of the earliest studies evaluating maintenance pemetrexed therapy in malignant pleural mesothelioma was reported by van den Bogaert et al., who retrospectively evaluated 27 patients without disease progression following pemetrexed-based induction chemotherapy, of whom 13 received maintenance pemetrexed. In that study, patients treated with maintenance pemetrexed experienced significantly longer time to progression (8.5 vs. 3.4 months) and overall survival (17.9 vs. 6.0 months) compared with those who did not receive maintenance therapy (*p* < 0.0001 for both). In our multicenter real-world cohort, maintenance pemetrexed was likewise associated with significantly prolonged progression-free survival (15.5 vs. 7.9 months, *p* < 0.001) and overall survival (22.3 vs. 16.4 months, *p* = 0.002). Although the earlier study was limited by a small and highly selected patient population and the lack of a randomized control arm, our findings derived from a larger, multicenter real-world dataset support and extend these results, suggesting that the survival benefit of maintenance pemetrexed may be applicable in routine clinical practice.

In contrast to these findings, the randomized phase II CALGB 30,901 trial, published in 2020, did not demonstrate a progression-free survival benefit for maintenance pemetrexed compared with observation in patients with unresectable malignant pleural mesothelioma who had not progressed after first-line platinum–pemetrexed chemotherapy^12^. Median progression-free survival was similar between the maintenance and observation arms (3.4 vs. 3.0 months, *p* = 0.973), and no statistically significant difference in overall survival was observed (16.3 vs. 11.8 months, *p* = 0.674). However, the study was limited by slow patient accrual and early termination, resulting in a relatively small evaluable population of 49 patients (27 in the maintenance arm and 22 in the observation arm), which substantially reduced its statistical power. These limitations may have contributed to the negative results and could partly explain the discrepancy between CALGB 30,901 and real-world studies, including our multicenter analysis, in which maintenance pemetrexed was associated with significantly improved survival outcomes.

More recently, Zeng et al. further supported the potential benefit of maintenance pemetrexed in malignant pleural mesothelioma in a retrospective cohort study published in 2022^13^. In this single-center analysis of 40 patients with unresectable disease who achieved disease control after first-line platinum–pemetrexed chemotherapy, maintenance pemetrexed was associated with a significantly prolonged progression-free survival compared with observation alone (median PFS, 8.5 vs. 3.0 months; *p* = 0.008). Although a numerical improvement in overall survival was observed in the maintenance group (26.4 vs. 15.7 months), this difference did not reach statistical significance. Similar to earlier reports, the study was limited by its retrospective design and small sample size; nevertheless, the consistent improvement in progression-free survival aligns with our findings. Notably, in our larger multicenter real-world cohort, maintenance pemetrexed was associated not only with a significant PFS benefit but also with a statistically significant improvement in overall survival, thereby extending the evidence for a clinically meaningful survival advantage in routine practice.

In our study, subgroup analyses based on best response to first-line platinum–pemetrexed chemotherapy were performed to further evaluate the effect of maintenance pemetrexed. Maintenance pemetrexed was associated with a significant prolongation of progression-free survival in both patients who achieved a partial response and those with stable disease compared with no maintenance therapy. Although the progression-free survival benefit appeared more pronounced in patients with a partial response, this difference is likely attributable to the larger number of patients in this subgroup rather than a true differential treatment effect. Importantly, the observation of a significant progression-free survival benefit even among patients with stable disease suggests that maintenance pemetrexed may provide clinical benefit beyond objective tumor shrinkage, supporting its use in patients with disease control after induction chemotherapy. At the last follow-up, of the 15 patients who were alive without disease progression after first-line therapy, 14 were receiving pemetrexed maintenance, supporting its potential role in maintaining disease control in real-world practice.

Beyond the impact of maintenance pemetrexed, several clinicopathological factors emerged as prognostic in our cohort, independent of maintenance treatment. For progression-free survival, epithelioid histology and best response to first-line platinum–pemetrexed chemotherapy were consistently associated with improved outcomes in both univariate and multivariate analyses. Patients with epithelioid tumors and those achieving a partial response experienced significantly longer progression-free survival compared with non-epithelioid histology and stable disease, respectively, underscoring the importance of tumor biology and depth of response in determining disease control duration.

In contrast, overall survival was influenced by a different set of prognostic factors. Consistent with the existing literature, epithelioid histology remained an independent predictor of improved overall survival in multivariate analysis^16^. Radiotherapy was associated with longer overall survival in univariate analysis; however, this association did not persist after adjustment in the multivariate model. Because detailed information regarding the indication, timing, and treatment intent of radiotherapy was not consistently available across all participating centers, this finding should be interpreted with caution. It is possible that the observed association may partly reflect patient selection and other unmeasured prognostic factors rather than a direct therapeutic effect of radiotherapy. Resectability was associated with overall survival in univariate analysis; however, this effect did not persist in multivariate analysis, indicating that the observed survival advantage is likely related to favorable baseline characteristics and patient selection rather than resectability itself. Notably, although best response to induction chemotherapy was a strong determinant of progression-free survival, it was not independently associated with overall survival, suggesting that post-progression disease course and subsequent treatments may mitigate its long-term prognostic impact.

Another important consideration is the biological heterogeneity of malignant pleural mesothelioma. Recent molecular studies have demonstrated that recurrent alterations involving BAP1, CDKN2A, and NF2 may influence tumor behavior, prognosis, and treatment responsiveness. In particular, BAP1 loss has been associated with distinct clinical outcomes and may affect sensitivity to systemic therapies, including chemotherapy and immunotherapy. Furthermore, molecular heterogeneity and differences in the tumor immune microenvironment may contribute to variability in treatment benefit among patients receiving maintenance strategies^17,18^. Because molecular profiling data were not routinely available in the present multicenter retrospective cohort, we were unable to evaluate treatment outcomes according to specific molecular subgroups. Future studies incorporating comprehensive genomic characterization may help identify patients most likely to benefit from maintenance pemetrexed.

The interpretation of our findings should also consider the evolving treatment landscape of malignant pleural mesothelioma. With the introduction of immune checkpoint inhibitors, particularly following CheckMate 743, immunotherapy has become an important treatment option for selected patients^8^. Therefore, the role of maintenance pemetrexed should be considered within the broader context of treatment sequencing. Future studies incorporating molecular and immunological biomarkers may help identify patients most likely to benefit from maintenance strategies and optimize treatment selection.

Despite the strengths of this multicenter real-world analysis, several limitations should be acknowledged. First, the retrospective design of the study introduces an inherent risk of selection bias and residual confounding, which cannot be fully excluded despite multivariate adjustment. In addition, because maintenance treatment was not randomly assigned and treatment decisions were based on physician discretion, the possibility that patients receiving maintenance therapy differed from those managed with observation in terms of unmeasured prognostic factors cannot be excluded. Although multivariable analyses and response-based subgroup analyses were performed, residual confounding and other biases inherent to retrospective studies may still have influenced the observed associations. Second, although the cohort of 96 patients represents a relatively large real-world population for malignant pleural mesothelioma, some subgroup analyses may still be underpowered. Third, treatment decisions regarding maintenance pemetrexed, radiotherapy, and surgical approaches were made according to institutional practices, leading to potential heterogeneity in treatment strategies. In addition, detailed information regarding the indication, timing, and treatment intent of radiotherapy was not consistently available across all participating centers. Therefore, the observed association between radiotherapy and overall survival should be interpreted with caution, as it may partly reflect patient selection rather than a direct therapeutic effect. Moreover, although post-progression treatment patterns were generally comparable between the study groups, the potential influence of subsequent therapies on overall survival outcomes cannot be completely excluded. Finally, detailed toxicity grading and patient-reported outcomes were not systematically collected, limiting a comprehensive assessment of treatment-related adverse events. Therefore, the findings should be interpreted with caution and should not be considered evidence of a causal relationship between maintenance pemetrexed and improved survival outcomes.

Overall, this multicenter real-world analysis adds meaningful evidence to the limited and conflicting literature on maintenance strategies in malignant pleural mesothelioma. Our findings suggest that maintenance pemetrexed may represent a clinically relevant option in selected patients achieving disease control after first-line chemotherapy, while also highlighting the prognostic importance of histology, performance status, and response to induction chemotherapy. Further prospective studies are warranted to better define patient selection and optimize maintenance treatment approaches in this setting.

## Conclusion

Maintenance pemetrexed following first-line platinum–pemetrexed chemotherapy was associated with significantly improved progression-free and overall survival in patients with malignant pleural mesothelioma who achieved disease control. These real-world findings suggest that maintenance pemetrexed may represent a feasible treatment option for selected patients who achieve disease control after first-line chemotherapy; however, prospective randomized studies are required to confirm these observations.

## Supplementary Information

Below is the link to the electronic supplementary material.

Supplementary Material 1

## Data Availability

The datasets generated and analyzed during the current study are available from the corresponding author on reasonable request.

## Abbreviations

BAP1: BRCA1 associated protein 1
CDKN2A: Cyclin dependent kinase inhibitor 2 A
MPM: Malignant pleural mesothelioma
NF2: Neurofibromin 2
PFS: Progression-free survival
OS: Overall survival
PR: Partial response
SD: Stable disease
EPP: Extrapleural pneumonectomy
P/D: Pleurectomy/decortication
