# Supplementary material for: Maintenance pemetrexed after platinum pemetrexed chemotherapy and survival outcomes in malignant pleural mesothelioma in a multicenter real world study
Source: Sci Rep. 2026 Jul 15;16:23876. doi: 10.1038/s41598-026-61625-0 (PMC13434598; doi:10.1038/s41598-026-61625-0)
Supplement: Supplementary file 1 [file 41598_2026_61625_MOESM1_ESM.docx]

Supplementary Information

Supplementary Table 1. Post-progression treatment patterns according to study group

| Treatment after progression | Maintenance pemetrexed (n = 40) | No maintenance pemetrexed (n = 41) |
| --- | --- | --- |
| Immunotherapy | 11 (27.5%) | 7 (17.1%) |
| Chemotherapy | 11 (27.5%) | 14 (34.1%) |
| Platinum–pemetrexed rechallenge | 6 (15.0%) | 3 (7.3%) |
| Best supportive care | 7 (17.5%) | 9 (22.0%) |
| Death before second-line therapy | 5 (12.5%) | 8 (19.5%) |
